# Supplementary material for: An Engineered sgsh Mutant Zebrafish Recapitulates Molecular and Behavioural Pathobiology of Sanfilippo Syndrome A/MPS IIIA
Source: Int J Mol Sci. 2021 May 31;22(11):5948. doi: 10.3390/ijms22115948 (PMC8197930; doi:10.3390/ijms22115948)
Supplement: Supplementary file 1 [file ijms-22-05948-s001.zip › ijms-1206088-supplementary.pdf]

Supplementary data for "An engineered sgsh mutant zebrafish recapitulates molecular and behavioural pathobiology of Sanfilippo syndrome A/MPS IIIA" – Douek *et al.* (2021), IJMS

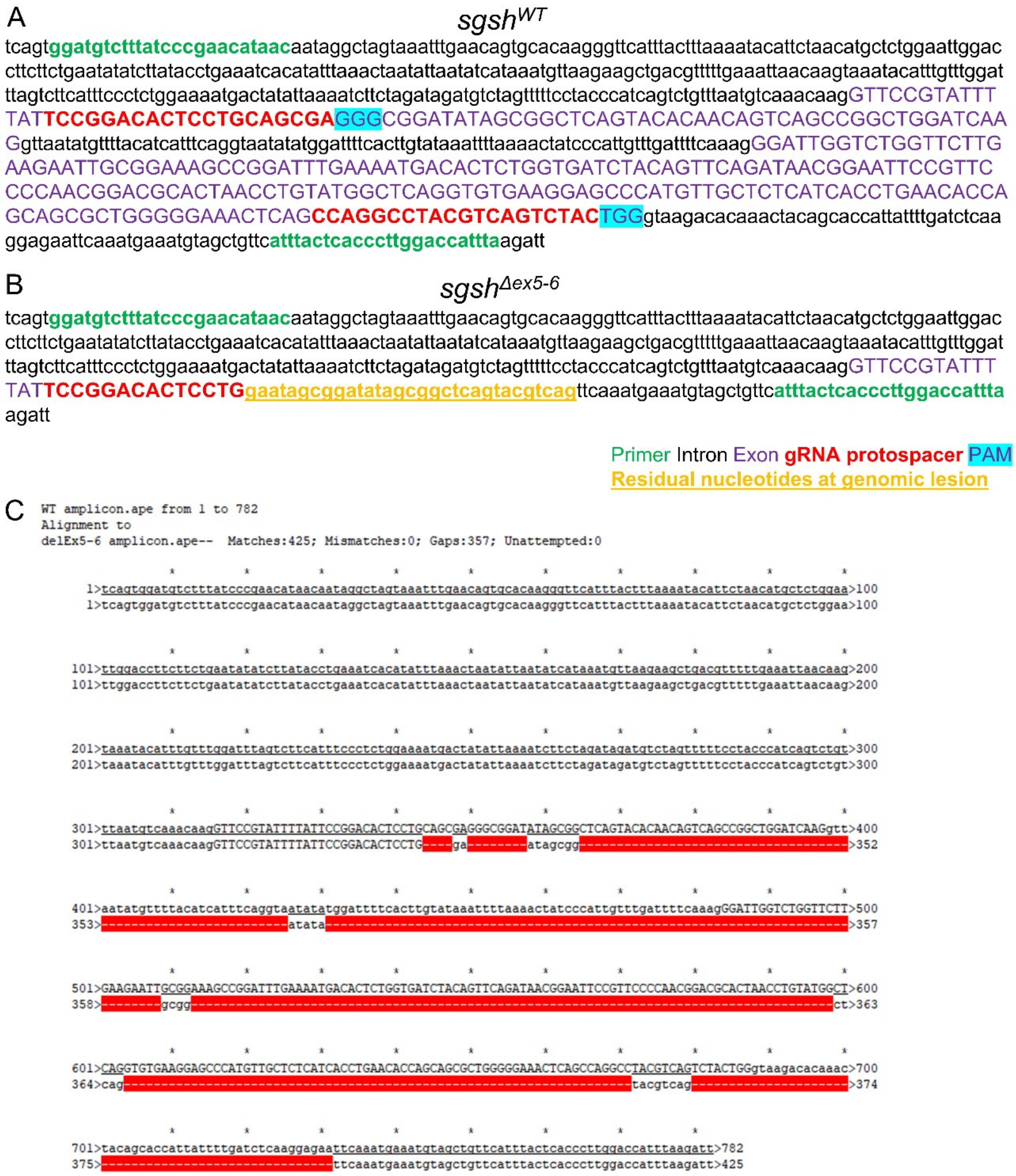

Figure S1. (A) Sanger sequencing product of wild type *sgsh* amplicon from PCR of genomic DNA.;**(B)** Sanger sequencing product of *sgsh*$^{\Delta ex5\text{-}6}$ amplicon from PCR of genomic DNA; **(C)** Alignment of wild-type and *sgsh*$^{\Delta ex5\text{-}6}$ Sanger sequencing products demonstrating that the residual nucleotides at the lesioned *sgsh*$^{\Delta ex5\text{-}6}$ locus are small fragments of the intervening region between the two gRNA target sites in the wild type locus.

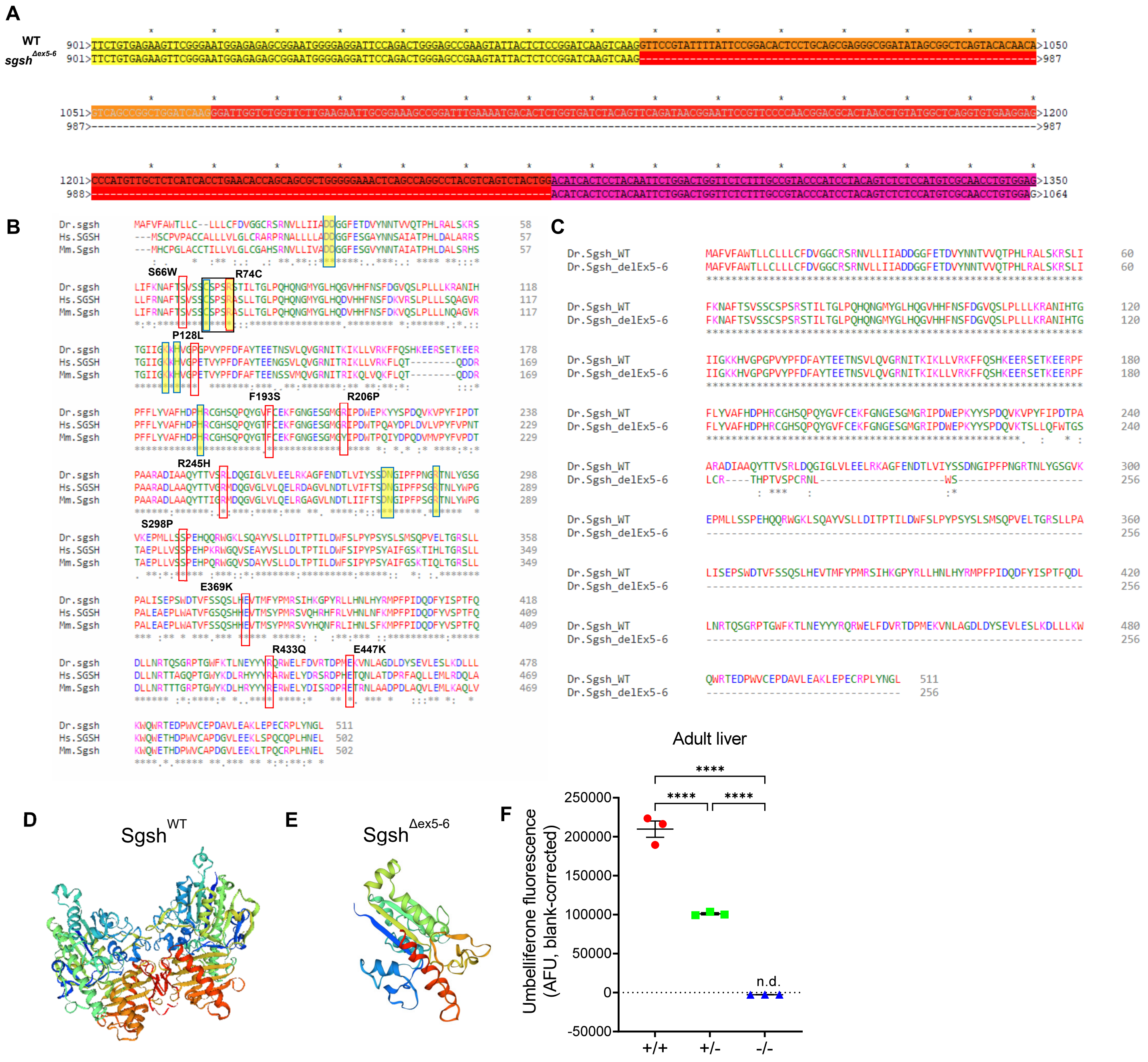

Figure S2. (A) Section of alignment from Sanger sequencing of wild type and *sgsh*$^{\Delta ex5\text{-}6}$ RT-PCR products of deleted region. Exon 4 is yellow, 5 is orange, 6 is red and 7 is purple; **(B)** Clustal Omega alignment of zebrafish (Dr.sgsh), human (Hs.SGSH) and mouse (Mm.Sgsh) amino acid sequences. Blue boxes with yellow highlighting indicate active site residues. Red boxes denote residues associated with pathogenic mutations in MPS IIIA patients, note strong conservation in zebrafish. Black box denotes the highly conserved C(X)PSR sulfatase motif; **(C)** Clustal Omega alignment of wild type zebrafish and Δex5-6 mutant amino acid sequences; **(D)** Modelling of wild type zebrafish Sgsh homodimer using SWISS-MODEL; **(E)** Modelling of Sgsh$^{\Delta ex5\text{-}6}$ monomeric protein product using SWISS-MODEL; **(F)** Enzymatic assay of Sgsh activity from total protein derived from adult liver from wild type, *sgsh*$^{\Delta ex5\text{-}6}$

heterozygous and homozygous zebrafish. Data presented as mean ± SEM and tested via ordinary one-way ANOVA with Tukey's multiple comparisons test; ****$p < 0.0001$.

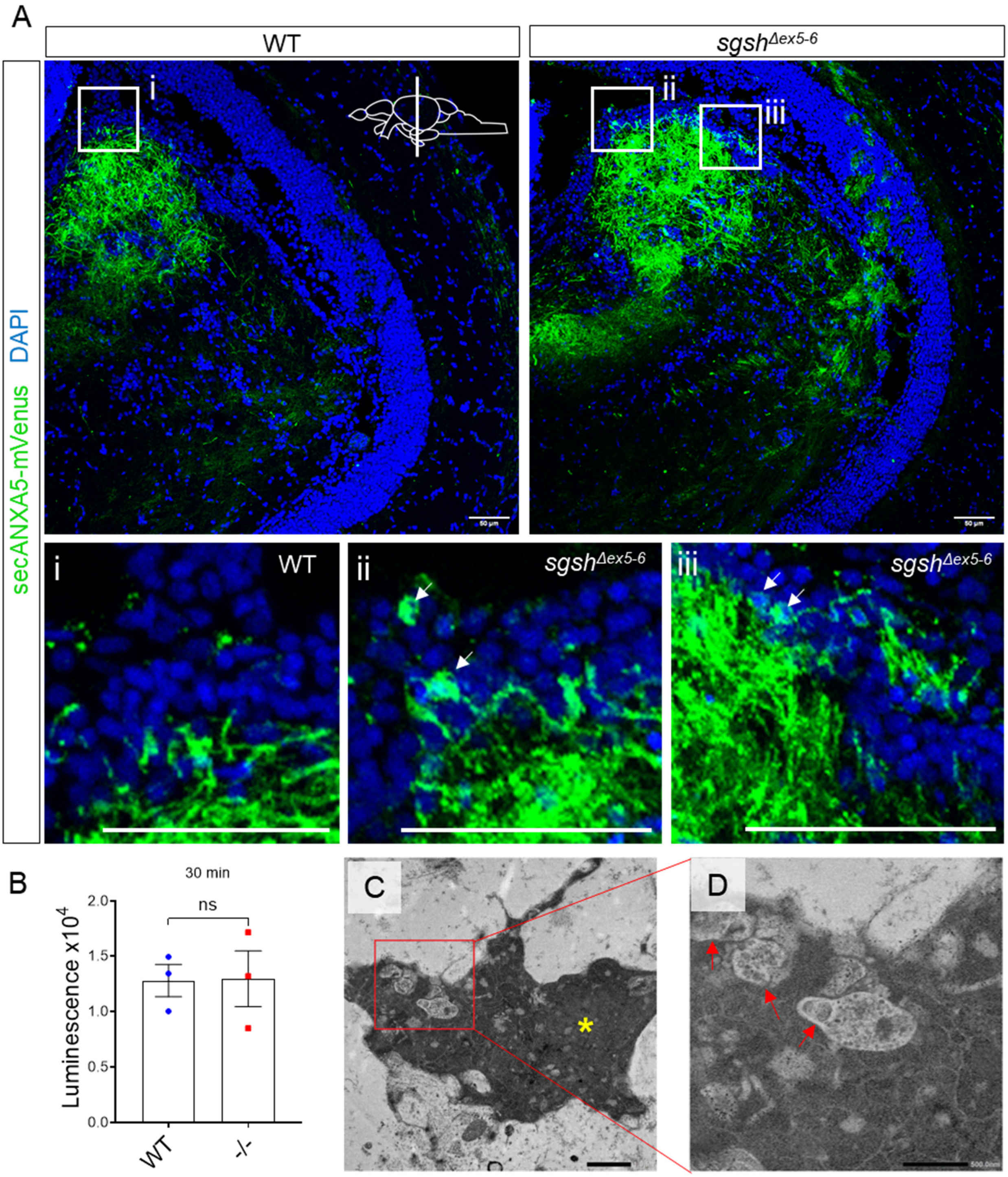

Figure S3. (A) The *Tg(-3.5ubb:SECHsa.ANXA5-mVenus)* transgene detects both myelin **(i)** and apoptotic cells **(ii-iii, arrows)** in adult brains due to the expression of phosphatidylserine, the target of transgene-encoded Annexin V, in both compartments. Scale bar 50 µm in all images; **(B)** Luminescence-based quantification of Caspase-3/7 activity in 50,000 dissociated cells from wild type or *sgsh*$^{\Delta ex5-6}$ brains. Data presented as mean ± SEM and tested by unpaired t-test. n.s. $p > 0.05$; **(C)** TEM micrograph of an apoptotic cell detected in an 18-month-old *sgsh*$^{\Delta ex5-6}$ brain,

in close association with activated microglia. Yellow asterisk indicates hyper-electron-dense apoptotic cell; **(D)** Zoom of boxed region in (**C**); red arrows indicate activated microglia as defined by amoeboid, migratory morphology and presence of small cytoplasmic granular corpuscles. Scale bars 1 μm in (**C**) and 500 nm in (**D**).
